# Advances in Molecularly Imprinted Polymers Based Affinity Sensors (Review)

**DOI:** 10.3390/polym13060974

**Published:** 2021-03-22

**Authors:** Simonas Ramanavicius, Arunas Jagminas, Arunas Ramanavicius

**Affiliations:** 1 Department of Electrochemical Material Science, State Research Institute Center for Physical Sciences and Technology (FTMC), Sauletekio av. 3, LT-10257 Vilnius, Lithuania; simonas.ramanavicius@ftmc.lt (S.R.); arunas.jagminas@ftmc.lt (A.J.); 2 Department of Physical Chemistry, Faculty of Chemistry and Geosciences, Institute of Chemistry, Vilnius University, Naugarduko 24, LT-03225 Vilnius, Lithuania

**Keywords:** immunosensors, affinity sensors, DNA-sensors, conducting polymers (CPs), biosensors, polymer-modified electrodes, electrochemical deposition, electrochemical sensors, electrochromic organic polymers, molecularly imprinted polymers (MIPs)

## Abstract

Recent challenges in biomedical diagnostics show that the development of rapid affinity sensors is very important issue. Therefore, in this review we are aiming to outline the most important directions of affinity sensors where polymer-based semiconducting materials are applied. Progress in formation and development of such materials is overviewed and discussed. Some applicability aspects of conducting polymers in the design of affinity sensors are presented. The main attention is focused on bioanalytical application of conducting polymers such as polypyrrole, polyaniline, polythiophene and poly(3,4-ethylenedioxythiophene) ortho-phenylenediamine. In addition, some other polymers and inorganic materials that are suitable for molecular imprinting technology are also overviewed. Polymerization techniques, which are the most suitable for the development of composite structures suitable for affinity sensors are presented. Analytical signal transduction methods applied in affinity sensors based on polymer-based semiconducting materials are discussed. In this review the most attention is focused on the development and application of molecularly imprinted polymer-based structures, which can replace antibodies, receptors, and many others expensive affinity reagents. The applicability of electrochromic polymers in affinity sensor design is envisaged. Sufficient biocompatibility of some conducting polymers enables to apply them as “stealth coatings” in the future implantable affinity-sensors. Some new perspectives and trends in analytical application of polymer-based semiconducting materials are highlighted.

## 1. Introduction

Affinity sensors are widely used in many analytical fields, but the most frequently they are applied for various biomedical purposes [1]. Due to the variation of different analytes and the variety of matrixes where these analytes are determined, many different analytical signal transduction techniques are applied to design suitable affinity sensors. Moreover, some new and advanced materials are used to improve the selectivity of affinity sensors, which is required to fulfil recent trends and requirements of newly designed analytical systems [2]. Due to significant efforts of researchers, biosensors are applied in the detection of many biologically active compounds in complicated biological aliquots such as blood, blood serum, saliva urine, etc. [3,4]. These sensors are used to solve some still challenging tasks in pharmacy and biomedicine, such as tissue regeneration [5] or sensor design [6,7]. To advance performance of sensors, various semiconductor-based structures are applied [8,9], very often such structures involve conducting polymers (CPs) and CPs-based heterostructures. CPs can be used for the formation of sensor-structures that are selective toward selected analyte, because they can be used for the immobilization of various biological materials, ranging from enzymes [10,11], antigens [12], antibodies receptors and for the formation of molecularly imprinted polymers [13,14]. CPs are characterized by a rather good electrical conductivity [15] and capacitance [16,17,18], adhesion to electrically conducting surfaces and mechanical stability [19,20], charge transfer ability, which can be successfully exploited in charge transfer from redox proteins towards metal- or carbon-based conductors [21]. Therefore, various electrochemically and optically active conducting polymers are used in the design of various sensors and biosensors as signal transducing systems.

Recently, many CPs are applied in sensor design, but among them the most frequently used are polypyrrole(Ppy), polyaniline (PANI) and polythiophene(PTH), poly(3,4-ethylenedioxythiophene) (PEDOT) [22,23,24,25,26].

Various methods can be applied for the formation of CP-based sensing structures, namely: chemical [27], electrochemical [16], enzymatic [10], and/or microorganism assisted [28,29,30,31]. The selection of the most appropriated monomers, which are forming backbone of CPs, is a critical issue in the design of sensing structures. Then, selected monomers are polymerized and, if necessary, formed CP-based layer can be very easily modified by entrapped DNA [32], redox proteins [33], antibodies [34] or other specific proteins [12], which are providing specific selectivity towards selected analytes. Hence, due to entrapped biological recognition elements different CP-based composite structures might have good selectivity towards analytes. Hence, the application of biologically active materials in affinity-based sensing devices (immunosensors, DNA-sensors, etc.) is often related to some disadvantages such as limited stability or expensiveness of applied biomaterials. Therefore, researchers are searching for some reliable replacements and are developing so called molecularly imprinted polymers (MIPs) [35] and some other types of “synthetic receptors”.

Taking in to account all above mentioned advantages of conducting polymers, we are aiming to overview the most reliable methods recently used for the formation of CP-based sensing structures, the application of conducting polymers and some other structures in the design of molecularly imprinted polymers in affinity sensors.

## 2. Oxidative-Chemical Polymerization Based Synthesis and Processing of Conducting Polymers

**Figure 1:**
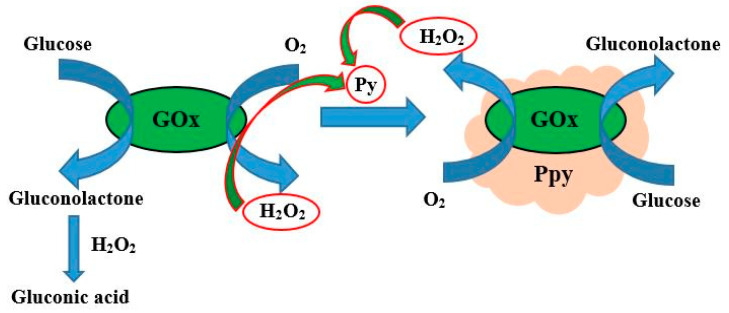
Formation of polypyrrole by glucose oxidase assisted polymerization, figure from reference [45].

**Figure 2:**
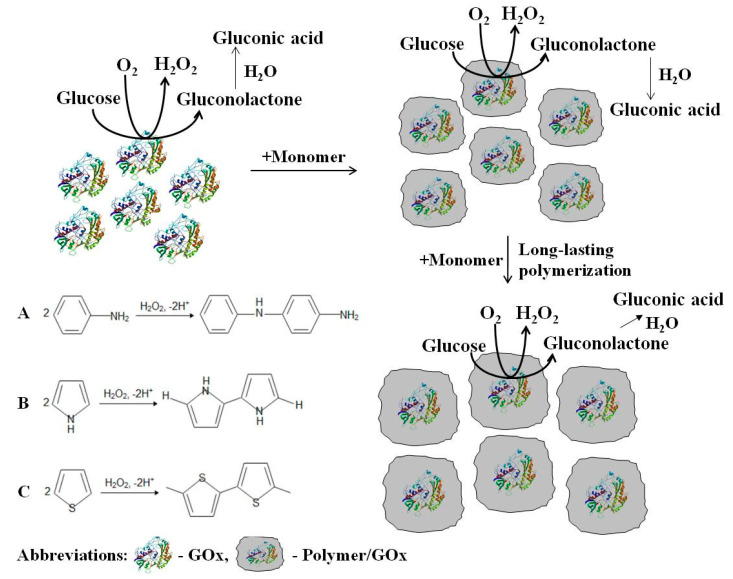
Formation of conducting polymer (**A**—polyaniline, **B**—polypyrrole, **C**—polytiophene) layers around redox enzyme–glucose oxidase, which during catalytic action is producing H_2_O_2_ that in here presented polymerization reactions is acting as an initiator. Adapted from [38].

Due to versatile technological applications, a variety of different methods for CPs synthesis have been developed. Sensing structures based on CPs can be formed by oxidative-chemical polymerization initiated by oxidizing compounds such as FeCl_3_, which was the most frequently used for the formation of conducting polymer–polypyrrole (Ppy). Some years ago, we have proposed to apply H_2_O_2_ as initiator/oxidator in the formation of polyaniline [36,37,38] (Figure 1 and Figure 2), polypyrrole [27,38,39] (Figure 2), polythiophene [38,40] (Figure 2), nanobiocomposite based on poly(1,10-phenanthroline-5,6-dione), poly(pyrrole-2-carboxylic acid) [41], poly-9,10-phenanthrenequinone [42], polyphenanthroline [21], carbazole [43], azobenzene [44] and some other conducting polymers.

**Figure 3:**
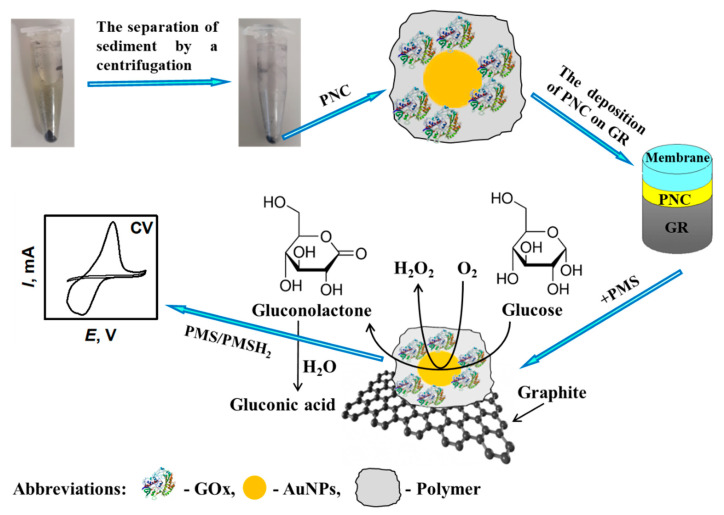
Principle scheme of formation composite structure consisting of polyaniline (PANI), gold nanoparticles (AuNPs) and glucose oxidase (GOx) PANI/AuNPs-GOx, which is followed by cyclic voltammetry-based investigation. Adapted from [56].

The advantage of H_2_O_2_ application is based on their ability to form pure Ppy—without any additives and/or dopants, because the excess of H_2_O_2_ can be easily degraded into water and oxygen, which both anyway are present in aqueous polymerization bulk solution. Hence, very pure polypyrrole, polyaniline, polythiophene can be formed, which mostly appears in the form of nanoparticles of different size when this polymerization method is applied. Enzymes and various nanostructures (e.g., gold nanoparticles (AuNPs)) can be embedded within CP-based particles formed [33,41,46,47,48]. Some our studies illustrated that polypyrrole-based structures have sufficient bio-compatibility towards stem cells [49,50] and do not irritate immune system of laboratory mice [51]. Due to simplicity of chemical polymerization significant amounts of CPs or CP-based hetero-structures can be synthesized, but it should be noted that due to limited solubility in such way only colloidal solutions of CPs can be formed. These CP-based colloidal nanoparticles can be modified by biological and/or other materials and then they can be applied for the formation of sensing layers of biosensors and/or other analytical systems. Very similar approach suitable for the synthesis of CP can be realized in the presence of some redox enzymes (oxidases, such as glucose oxidase and many other oxidases) and their substrates because during catalytic action of oxidases hydrogen peroxide is formed [10,33,41,46,47,48], which as it was noticed before, can be involved in the synthesis of some conducting polymers, namely Ppy [10,33,41,46,47,48,52], PANI [37,38,46,47,48], polythiophene [38,40], poly-9,10-phenanthrenequinone [42], polyphenanthroline [21] and some other CPs (Figure 3). Enzymatic formation of CPs is performed in water-based environment, at neutral pH and temperatures that are close to “room temperature” because under these conditions maximal enzymatic activity is observed [53]. For here mentioned enzymatic synthesis glucose oxidase, which was dissolved in water [46,48] or immobilized on electrode [10,33,41,47], was employed and it is very interesting and purposeful that the enzyme molecules are encapsulating them-self within formed CP shell during the synthesis, which is performed. Conducting polymers formed by this way show sufficient biocompatibility towards enzymes [33,41,46,47]. Catalytic activity of encapsulated enzymes is retained when enzymes are encapsulated in such CP-based matrixes, but catalytic characteristics of such CP structures based on encapsulated enzymes are different from those of native enzymes. These differences are induced by formed CP-layer, which hinders the diffusion of reaction substrates towards enzyme active site as well as diffusion out of formed reaction products. Therefore, in this way formed CP-based nanoparticles and other structures formed on the surface of electrode are well suitable for the design of biosensors [54,55] and some bioelectronics based devices.

In addition to the application of H_2_O_2_ [27], we have developed polypyrrole synthesis method where [Fe(CN)_6_]^3−^ was applied as an initiator of polymerization reaction [57]. This approach is very useful, because if such polymerization reaction is performed in the presence of microorganisms, during the subsequent initiation of polymerization reaction forming [Fe(CN)_6_]^4−^ can be recycled by the re-oxidation of this compound by redox enzymes and/or metabolic redox-processes, which are taking place in life-cycle of microorganisms [28,29,30,31].

Chemical synthesis enables to synthesize micro- and nano-particles of conducting polymers that could be useful for the formation of MIP-based structures required for sensors, affinity chromatography [58] and some other technological purposes [59]. When the synthesis of CPs is completed, spin-coating techniques [60] can be applied to deposit polymer layer on the surface of signal transducer.

## 3. Advantages of Electrochemical Conducting Polymer Based Layer Formation

**Figure 4:**
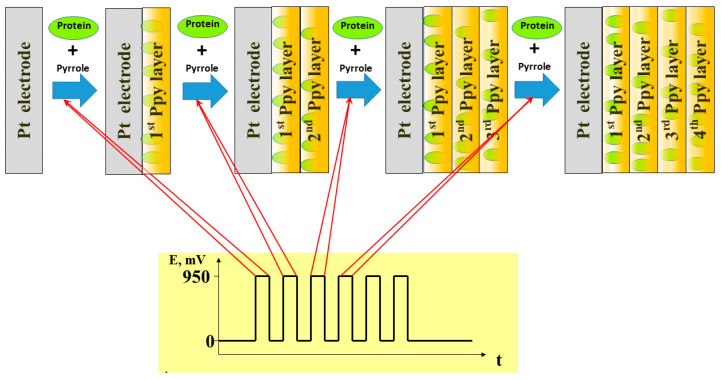
The scheme of Ppy electrochemical deposition by potential pulses and entrapment of proteins within the formed Ppy layer, figure from reference [45].

Conducting polymers are poorly soluble in usual solvents, for this reason it is not very easy to apply CPs in the formation of sensing layers (Figure 4). These technological problems can be solved by electrodeposition, which is more reliable for the formation of CP-based structures on conducting substrates. The selection of electrodeposition methods [61] and the adjustment of parameters that are used during the deposition of CP-based films enables to form sensing layers with very different analytical characteristics. The most easily adjustable synthesis parameters are: (i) voltage of applied potential, (ii) the duration of potential pulses or potential sweep rate used when potential cycling is applied, (iii) the limitation of electrical current passing through the electrochemical system, [62,63], (iv) some other additionally applied external factors (e.g., treatment by ultrasound) [64]. The variation of all these parameters enables changing many physicochemical properties of polymeric layers. Hence, some electrochemical characteristics of CP-based layers can be adjusted by the adaptation of concentrations of all materials, which are used in polymerization bulk solution [65,66,67]. The most important characteristics including sensitivity and linear range of CP-based sensors are predetermined by the thickness, density, permeability and other properties of CP-based layers. Therefore, by variation of above mentioned and some other polymerization conditions (such as thickness and morphology) the porosity of deposited conducting polymer layer can be easily changed [12,68]. Control of formed layer morphology enables to change the permeability of CP-based films [12,69]. The diffusion of target/analyte and some other compounds through CP-based matrix is very important for the action of affinity sensors based on these structures. Conducting polymers from this point of view are very attractive, because by the selection of proper synthesis conditions porous structures based on CPs can be formed [70]. In addition, such porous structures mostly are amorphous and do not display long-range order of polymer-film forming molecules. Some researchers are reporting the possibility to adjust the porosity of CPs by using some organic compounds as spacers, which are interlinking different polymer chains [71]. Conducting polymers of high porosity were exploited in the design of sensor dedicated for the determination of antibiotic-aminoglycoside, which was evaluated in aqueous samples [72]. Hence, electro-deposition of CP-based structures offers many possibilities for the design of sensors with tunable analytical characteristics. In addition to above mentioned advantages, there are many other serious reasons to choose electropolymerization for the formation of CP-based layers, because: this technique is much faster than the classical oxidative-chemical polymerization in the bulk but also it can be carried out in situ on the working electrode’s surface [73], and if potentiostat/galvanostat is controlled by properly developed software, then the whole process can be clearly observed on computer screen and evaluated/controlled using elaborated mathematical algorithms [12], The most recently used electrochemically deposited polymers are: polyaniline [36,56], polypyrrole [1,12,16,19,22,23,35,39], polythiophene and poly(3,4-ethylenedioxythiophene) (PEDOT) derivatives [36,74], Poly-9,10-phenanthrenequinone [42], In addition, some derivatives of these polymers can be electropolymerized and/or electro-copolymerized with some other monomers, which themselves are also forming conducting polymers, e.g., polypyrrole was copolymerized with other polypyrrole derivatives containing different 4-(pyrrol-1-yl)-benzenethiol groups and such copolymer was showing some properties desired for the application in sensors and biosensors [75].

Both, chemical and electrochemical polymerization can be applied for the development of electrochemical affinity-sensors based on MIPs. However, electro-polymerization has more advantages [76] in comparison to chemical methods. As it allows many more possibilities for the control of morphology, thickness and doping of electrochemically formed MIPs. Moreover, some additional electrochemical manipulations are possible after the formation of initial electrochemically deposited polymer layer, e.g., some conducting polymers such as polypyrrole can be overoxidized by applying higher electrode potentials in comparison with that, which are required for the formation of corresponding conducting polymer. The same overoxidation can happen in the presence of oxygen, which is dissolved in polymerization bulk solution, and/or by oxygen formed at the anode by the oxidation of hydroxyl ions.

From one side, the overoxidation is an unwanted process, because it can terminate polymerization process and/or damage π-π conjugated system of conducting polymers, but during the formation of MIPs this process plays a positive role since it creates oxygen containing groups (mainly carboxyl (–COOH), carbonyl(–CH=O), hydroxyl (–OH)) in close proximity to entrapped molecules and these charged groups form specific environment, which is suitable for the recognition/attachment of imprinted template molecule the same that after the formation of MIP acts as a target. Hence, electrochemical polymerization, which is followed by overoxidation is a powerful combination of electrochemical technique, which can be used in the development of new conducting polymer-based MIPs. It should be noted that chemical oxidative polymerization is simple and suitable for the production of significant amounts of molecularly imprinted conducting polymers [77,78]. During the development of electrochemical affinity sensors, electro-polymerization shows significant advantages in comparison with oxidative chemical polymerization, because this electrochemical method enables the deposition of molecularly imprinted conducting polymer-based film on the electrode surface. Electropolymerization can be conducted in solutions of different composition and concentration of monomer, which is required for the formation of MIP, and/or template molecules. Variation of electrical parameters during electrochemical polymerization opens a very attractive possibility for the tailoring of some physical properties of deposited polymers. The most attractive properties to be tailored are: thickness, conductivity, morphology, and homogeneity. In addition, the oxidation level of deposited MIP-based film can be easily controlled by the selection of particular electrochemical method and adjustment of applied potentials, their durations and many other parameters applied for electopolymerization. For electrochemical formation of conducting polymer based MIPs potentiodynamic methods are providing the most reliable sensing layers, because the variation of potential enables control different phases of conducting polymer based film formation, e.g., during the polymerization phase, which is performed at high potential concentration of polymerizable monomer and template molecule is decreasing and therefore some time is required for the ‘system relaxation’ and reestablishment of concentration of polymerizable monomer and template molecule at the surface of electrode. If overoxidation phase is applied to form conducting polymer-based MIP, this process can be easily controlled. It should be taken into account that in some research serious problems during the removal of imprinted target molecules have been observed. However, in some cases, these problems were easily resolved by over-oxidation of formed MIP [79], which shows additional advantages of over-oxidation process.

**Figure 5:**
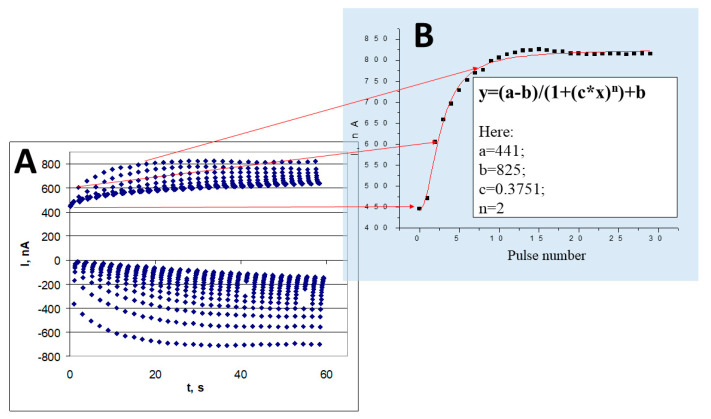
(**A**) Chrono-amperograms, registered during electrochemical deposition of polypyrrole by potential-pulse mode. (**B**) Dependence of anodic peaks on the pulse number during electrochemical deposition, figure adapted from reference [12].

Development of molecularly imprinted over-oxidized polypyrrole-based sensors is probably the most efficient direction in MIP-related area, since Ppy-based layers can be electrochemically deposited from aqueous solutions of pyrrole while using simple electrochemical methods [12,23,35,80], which can be well controlled using developed mathematical algorithms [12] (Figure 5). In many studies, electrochemically synthesized Ppy-based MIPs were designed and applied in sensors. These sensors were suitable for the determination of analytes with low molecular weight, namely: caffeine [23,81], theophylline [35,82], dopamine [83,84], histamine [85], gallic acid [86], quercetin [87], sarcosine [88], bilirubin [89], microcystin-LR [90], tetracycline [80], adrenaline [91], sulfanilamide [92], uric acid [93], ganciclovir [94], L-aspartic acid [95], serotonin [96], kanamycin [97], cysteine enantiomers [98], fenvalerate [99], dibutyl phthalate [100] and testosterone [101]. In our previous study we have electrochemically deposited caffeine-imprinted Ppy layer on a quartz crystal based resonator and applied this resonator in a quartz crystal microbalance based sensor [23]. We have observed that during the interaction between dissolved caffeine and MIP-based layer the equilibrium is shifted toward the formation of caffeine/MIP-complex. When the interaction of caffeine-imprinted MIP with dissolved theophylline was investigated opposite result was obtained and the formation of caffeine-MIP complex with theophylline was almost imperceptible. Hence, formed caffeine-MIP showed significantly better selectivity toward caffeine in comparison with that toward theophylline, which is a homologue of caffeine. Electrochemical affinity sensor based on hyaluronic acid and multi-walled carbon nanotubes additionally modified by tryptamine-imprinted polypyrrole-sulfonated graphene were developed [102]. This sensor well discriminated tryptamine from several others in this research tested interfering materials such as (dopamine, tryptophan and tyramine). One more molecularly imprinted polypyrrole-based affinity sensor was developed for the determination of epinephrine, which was within Ppy deposited on glassy carbon electrode pre-modified by multi-walled carbon nanotubes and silica nanoparticles [103]. In this sensor, multi-walled carbon nanotubes and silica nanoparticles provided multi-porous network structure, which increased the accessibility of analyte (epinephrine) towards imprinted sites. Screen printed carbon electrodes were modified by clopidol-imprinted Ppy structures, which were electro-deposited from water-based solution containing both pyrrole and clopidol [104]. For the determination of analytical signal by this sensor differential pulse voltammetry was applied. Polymer based on NO^3−^-imprinted phenothiazine derivative, poly(Azure A), was applied as nitrate scavenger in aqueous contaminated environments [105].

MIPs-based on ortho-phenylenediamine (o-phenylenediamine) [106] and some other phenylenediamine-derivatives [107,108] are frequently applied for analytical and pharmaceutical purposes, e.g., molecularly imprinted ortho phenylenediammine (o-phenylenediammine) was applied for the determination of butyrylcholinesterase [109] and anticancer drug pemetrexed [110]. Therefore, MIPs are very attractive for the determination of various anticancer drugs [111]. Erythromycin imprinted poly-meta-phenylenediamine was electrochemically deposited on screen printed electrodes and was applied for selective determination of erythromycin in real aqueous samples [108]. Ratiometric electrochemical sensor based on polythionine modified by corresponding MIP was applied for the detection of dopamine [112]. Sulphanilamide imprinted polyresorcinol electrochemically deposited on a gold electrode was applied for the determination of antibiotic sulphanilamide in water and milk samples [113]. Azorubine imprinted poly(1-naphthylamine), triphenylamine based copolymer was applied for the determination of azorubine in water samples [114]. Electrochemically polymerized nicotinamide was imprinted by dopamine (DA) and chlorpromazine and deposited on a gold electrode modified by graphene oxide-based quantum dots and applied for electrochemical synthesis of both imprinted compounds [115]. It was demonstrated that some metal oxides (such as TiO_2_) can be also imprinted by some proteins, e.g., potentiometric urea biosensor based on TiO_2_ layer molecularly imprinted by urease was developed [116].

## 4. Physicochemical Properties of Conducting Polymers

Conducting polymers are based on π-π conjugated bond structure formed along polymeric chain, therefore, π-electrons are delocalized along these conjugated bonds and, for this reason, CPs are electrically conducting [21,68,117,118]. In addition, CPs have advantageous electrochemical [21], optical [119] and many other physical properties that can be exploited for technological purposes [33,119,120]. For this reason, many CPs are applied in the design of various devices such as rechargeable batteries and ‘smart windows’ that are changing transparency and translucency towards passing light, electrochromic displays, organic-photovoltaics and light emitting diodes for organic-electronics, sensors and biosensors [121,122,123]. Some of these CPs have very versatile electric/optical [124], affinity [125], and/or electrochemical [10] properties and, therefore, variables of some physicochemical properties such as electrical impedance, capacitance, optical density, etc. It is important that the variation of photoluminescence or other optical properties can be exploited for the registration of analytical signals [126,127]. Among a vast variety of different CPs, polypyrrole (Ppy) is one of the most frequently used conducting polymers in the design of affinity sensors [10]. Some nanocomposite structures based on conducting polymers (e.g., SWCNTs/PANI-based hetero-structure) have been applied in the design of sensors suitable for the detection of Pb^2+^ [128], Hg^2+^, Cu^2+^ [128,129] and some other heavy metal ions [130]. Due to high affinity towards metal ions and other hazardous compounds, some CPs-based structures can be applied in chromatography and/or for the extraction of such materials from polluted/intoxicated environments [131].

Some conducting polymers are cheap and have good environmental stability and easy tunable physical properties [10]. Besides, CPs can form various heterocomposites with inorganic [33,41,46,47,48], organic [132], and biomaterials [38,46,48]. Many of above mentioned electrochemically active CPs are well suitable for immobilization of biomaterials [133,134], therefore, they have been used as transducers in sensors [135].

Efficient doping of CPs can increase electrical conductivity of CPs by several orders of magnitude [136]. Electrical conductivity of polypyrrole (Ppy) [12], polyaniline (PANI) [137], and poly(3,4-ethylenedioxythiophene)/poly(styrenesulfonate) (PEDOT/PSS) [123] is based on p- or n-type charge carriers induced by corresponding dopants. Doping/de-doping by some materials is reversible and induces well detectable changes of electrical and optical properties, therefore, it can be applied for the design of some affinity sensors in which specific affinity of conducting polymers towards some ions is exploited [137]. In addition, the conductivity of CPs can be changed by electrochemical and/or chemical oxidation/reduction. Electrochemical sensors based on Ppy and PANI are operating at ambient conditions and have sufficient sensitivity, therefore, these CPs were successfully applied in sensing elements of electrochemical sensors.

Due to attractive optical properties conducting polymers can be used in optical sensors [138,139]. Conducting polymers can be well exploited in the design of photoluminescence sensors [140,141]. Several carbazole-based conducting polymers (*N*-benzylcarbazole, *N*-benzyldimethoxy-carbazole and *N*-benzyldibromo-carbazole) were used for the development of photoluminescence sensors suitable for the detection of such pesticides as: isopropalin, trifluralin, imidacloprid, fenitrothion, cyfluothrin and glyphosate [120].

In contrary to photoluminescence ability of some conducting polymers [120,140,141], our research team has determined that some other conducting polymers (e.g., polypyrrole) have a very attractive feature to quench photoluminescence of adsorbed molecules, which are exhibiting photoluminescence [142,143]. This effect is based on so called “Forster resonance energy transfer (FRET)” that can be observed if photoluminescence emitting molecules are located in close proximity to photoluminescence quencher. This property of CPs can be well exploited in the design of photoluminescence-based immunosensors, where CP-based matrix is used for the immobilization of biomolecules that are exhibiting biological recognition and for the quenching of photoluminescence of various not specifically adsorbed compounds (e.g., proteins), while photoluminescence exhibiting target molecules immobilized on conducting polymer are located far out of the distance at which FRET is still efficient [142,143]. Therefore, such systems enable increasing both selectivity and sensitivity of some affinity-sensors [142,143]. In several investigations, we have shown how Ppy-based structure can be applied for the reduction of the photoluminescence, which is generated by non-specifically bounded interfering materials, during the registration of sensor response [142,143]. Protein–ferritin–molecularly imprinted poly-scopoletin microarray has been formed by microelectrospotting on bare gold-based surface plasmon resonance (SPR) imaging chips for the determination of ferritin [144].

Electrochromism is another important opto-electrochemical characteristic of many CPs that can be well adapted for the design of affinity sensors [137]. Electrochromic effect is a reversible variation of some optical characteristics if material is oxidized or reduced by applied electrical potential/current and materials that are changing their visible color are considered as electrochromic, therefore, some such materials like metal oxides (WO_3_ [145]) and conducting polymers (PANI [137], Ppy [39] PEDOT/PSS [137] etc.) can be applied for the design of sensors. Some such materials have more than two oxidation states that are differently colored, therefore they are called as ‘multi-electrochromic’ materials. Some electrochromic materials can be incorporated in structure of various devices, which enables the modulation of their optical transmittance, absorbance, light emission, and/or reflectance. The most efficient electrochromic compounds have distinct differences of optical characteristics at differently colored states, fast transition from one state to another and good durability. Some gaseous materials, volatile organic compounds, vapors and various dissolved materials are changing electronic structure of the electrochromic material, therefore, variations of their spectra are observed. In addition, some semiconducting properties of these compounds can be changed, therefore, simultaneously different measurements (optical and electrical) can be applied, e.g., for optical/electrochemical determination of Cu(II) ions [137]. Electrochromic conducting polymers can be applied for the design of optical sensors dedicated for the determination of some gaseous materials (e.g., CO_2_ and NH_3_) that are water soluble and are changing the pH of solution where transparent ITO based electrode additionally coated by electrochromic material is immersed [74,146,147]. Therefore, Ppy, PANI, and PEDOT/PSS, and various composites based on these CPs are often used in the design of electrochromism-based sensors. Electrochromic and some other optical properties of CPs depend on many factors including application of various dopants [137,148], which can increase the application of electrochromic sensors for the determination of new analytes.

Various artificial polymers can selectively recognize biocompounds and are more resistant to harsh physical, chemical, and physiological conditions than natural biopolymers. Therefore, due to advanced stability and recognition of analytes at a molecular level, MIPs are powerful tools for the development of next-generation chemical sensors [149]. Therefore, due to relatively low cost, easy preparation and good stability MIP-based sensors have great potential for practical applications and commercialization [150]. The basis for MIP formation and action was proposed on the basis of phenomenological thermodynamic model for the chemo-responsive shape memory effect in polymers based on Flory-Huggins solution theory [151]. This model predicts the constitutive relations and working mechanism of the chemo-responsive shape memory effect in shape memory polymers. On the origin of the Hildebrand solubility parameter [152,153], Flory-Huggins interaction parameter [154] and polymer relaxation theory, a phenomenological model has been proposed, which enables to quantitatively identify the factors influencing the chemo-responsive shape memory polymers. A free-energy function can be implemented to couple the constitutive relations of the chemical potential and stress as a function of the weight fraction of solvent and stretch, respectively. Furthermore, the simulation of the phenomenological thermodynamics model can be compared with the available experimental results and the simulation results of a semi-empirical model reported in the literature for verification. Swelling degree [155], swelling effect-induced shape recovery and complex shape memory behavior [156] are also very important characteristics, which should be taken into account during the development of polymers with chemo-responsive shape memory effect. Recently, to predict and/or optimize the efficiency of newly designed MIPs various computational methods based on DFT [157,158] and molecular dynamics [159] are applied. Hildebrand’s [152,153] and Hansen’s [160] theories for the prediction of polymer compatibility with porogenic solvents are used to predict the efficiency of MIP performance in different solvents, as it was well demonstrated in the case of L-phenylalanine imprinted within macroporous poly(2-aminoethyl methacrylate-co-2-hydroxyethyl methacrylate-co-ethylene glycol dimethacrylate) [161].

## 5. Entrapment of Proteins within Polymer Layers during the Development of Affinity Sensors

A large number of affinity sensors based on immobilized proteins have been developed, they most commonly are called as affinity sensors. An immunosensor is a type of affinity sensor in which a specific target analyte (e.g., antigen (Ag)) is detected by formation of immune complex between antigen and immobilized antibody (Ab) what results in the generation of a measurable response [162]. In affinity sensors CPs can serve as: (i) matrix for the immobilization of antibodies, receptors or other compounds that are able selectively bind analyte [12]; (ii) parts of signal transducer where the variation of semiconducting properties of CPs can be exploited for the generation of analytical signal [1,12,21], and (iii) molecular imprint-based structures that are recognizing imprinted targets [93,125,163,164]. Electro-deposition enables to form sensing layers that are containing proteins entrapped within the polymer layer (e.g., antibodies, receptors) or antigens, which are able to bind selectively some specific antibodies that are present in the sample) [12]. We have demonstrated that due to remarkable electrochemical capacitance conducting polymer-based films are amplifying electrochemically registered analytical signals of immunosenors, especially if these signals are generated using potentiodynamic electrochemical approaches [12]. Oriented immobilization of affinity agents is very important for the development of all kinds affinity sensors [142,165,166], because some analytes, which are usually determined by affinity sensors, are large and cannot freely-diffuse within CP-based matrix and/or other materials based sensing layers, therefore, they can efficiently bind corresponding sites only in the cases if they are properly exposed towards solution [93]. Therefore, the selection of proper immobilization methods is critical during the development of affinity sensors. A number of electrochemical affinity sensors suitable for the determination of pesticides such as paraquat were designed [167]. Hence, the correct orientation of immobilized antibodies [168], selected fragments of antibodies [165], and receptors [166] increases the efficiency of affinity sensors. A capacitive immunosensor based on o-phenylenediamine electrochemically deposited on indium tin oxide glass (ITO) electrode has been developed, after the deposition o-phenylenediamine layer and modification by bonding with anti-sulphathiazole antibody. Such sensor has been applied for the determination of sulphathaizole in spiked drinking water and milk by electrochemical impedance spectroscopy [169].

## 6. Formation of MIPs Imprinted by Proteins and by Other Large Biological Objects

Many conventional immunoanalytical techniques provide ability for accurate determination of various analytes. However, mostly these techniques such as enzyme linked immunosorbent assay (ELISA) are requiring expensive immune-chemicals and/or long-lasting analyte determination protocols and are based on the application of expensive and sophisticated equipment. Therefore, many research efforts are dedicated to the replacement of antibodies, receptors, many other affinity exhibiting proteins and DNA-based structures (e.g., DNA-aptamers) by artificial receptors or MIP-based structures. Due to this fact, the development of affinity sensors based on molecularly imprinted polymers (MIPs) has been achieving significant attention as a new trend of sensorics. Sometimes MIPs are determined as “biomimetic receptors”, which are formed by the polymerization of corresponding monomers in the presence of the analyte, which is acting as a template for the formation of molecular imprints [23,35,82,85,93,125,170]. After the removal of template three dimensional imprints are formed within imprinted polymer-based matrix. Such MIP-modified polymer has not only the shape and dimensions suitable for analyte binding but also provides complementary electrostatic environment that is optimal for the recognition of an imprinted analyte. Hence, these imprints are complementary to removed template. Therefore, such artificially created cavities are very selectively recognizing imprinted molecules and the action of MIPs is similar to that of antibodies or receptors. In addition, MIPs-based sensors are rather stable, because they mostly are based on a stable polymeric-matrix, e.g., acrylamide [171], acrylic acid and methacrylic acid, which both are frequently applied in the design of various molecularly imprinted polymer-based structures [172,173,174,175]. For the development of MIPs many different methods can be applied. However, most of these methods have some similar development/application phases: (i) formation of MIPs very often starts from the pre-incubation of polymerizable monomers, which are modified with the attached functional groups that are able to recognize and to bind the particular group of used template molecule; (ii) monomers, which are able to cross-link polymeric structure but do not have any attached functional groups, added into polymerization bulk solution and where in the presence of corresponding initiators and/or physical stimulation, the co-polymerization is performed; (iii) imprinted template molecules are removed from the polymer matrix and MIP-based structure is formed [176]. MIPs for the determination of large organic analytes such as Δ4-androstene-3, 17-dione, 1, 4-androstadiene-3, 17-dione, testosterone, testosterone propionate, β-estradiol, progesterone, were designed [174]. In these sensors, methacrylic acid was used as a matrix, which was successfully imprinted by above mentioned templates, and used in affinity-sensor design. One more electrochemical affinity-sensor for the determination of β-estradiol based on molecularly imprinted bifunctional monomers, *N*-phenylethylene diamine methacrylamide, has been reported in another research [175]. Another MIP, which was selective to β-estradiol, was applied for the modification of Fe_3_O_4_-based magnetic nanoparticles that were also applied in MIP-based sensor design [177]. Estradiol was imprinted within electrochemically deposited overoxidized polypyrrole [178]. Molecularly imprinted poly(ethylene–*co*–vinyl alcohol) heterocomposite with quantum dot nanoparticles was applied for optical determination of salivary proteins [179], and in many other researches some MIPs in different with nanomaterials were applied for electrochemical/optical assays [180]. Copper-based metalorganic framework molecularly imprinted by tetrabromobisphenol A was designed by a sol-gel method and it showed some enzyme-like activity towards oxidation of tetrabromobisphenol A in the presence of hydrogen peroxide [181].

**Figure 6:**
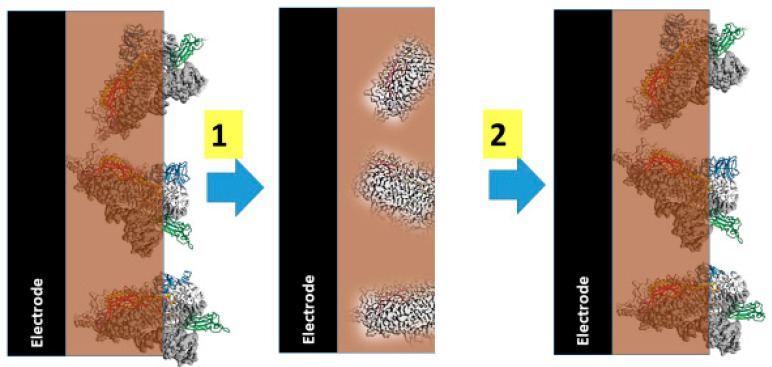
Representation how protein imprinted MIP sensor sensors are designed and are acting, 1—extraction of imprinted proteins, 2—action of MIP based sensor in the solution containing similar proteins. Adopted from [2].

Molecular imprinting of proteins is a very attractive research direction [182] because expensive and unstable biological compounds such as antibodies [12] and receptors [166] can be very efficiently replaced in various immunanalytical systems by MIP-based structures [183]. However molecular imprinting of proteins is not very trivial and is related to several critical challenges [184] such as extraction of proteins from MIP-matrix and multiple reusability of such imprinted protein-based sensors (Figure 6) [2,185], conformational changes of proteins during imprinting phase [186], and suitable orientation of proteins during the imprinting phase [187]. Hence, due to numerous efforts of various research groups, significant progress has been achieved in the development of MIP-based sensors for the determination of proteins, which sometimes are called as “plastic antibodies” [188,189], “artificial receptors” or “synthetic receptors” [2,185,190]. During this development many practical problems traditionally associated with molecularly imprinted polymers (MIPs), should be solved, that includes some challenges related to imprinting of proteins, namely, hydrophobic nature of some polymers that are applied for the formation of MIPs, insufficient compatibility with template, and the formation of not-specific binding regions in imprinted polymers that are responsible for non-specific binding of different proteins and/or other molecules. The success in MIP-formation is well related to technological advances in organic chemistry, polymer chemistry and nanotechnology [2,185,191]. According to here presented challenges, which are facing technologists during the development of MIPs, it is evident that this promising research direction, which enables to develop MIPs as a real alternative to antibodies and/or receptors, is based on mulitidisciplinary and interdisciplinary investigations. Despite impressive number of recent publications related to the development of MIPs for various analytes, commercial applications of these promising materials are still limited and many above mentioned problems need to be solved to overcome critical limitations before they will find their place in commercially available bioanalytical systems [2,192].

It is important to note that conducting polymers can be molecularly imprinted by some high molecular mass biomolecules including DNA [2,125] and proteins [2,185,193,194]. During the development of DNA sensors conducting polymer–polypyrrole—was applied for both immobilization of single stranded DNA (ssDNA) and detection of complementary strand of ssDNA [32], and for the formation of molecular imprints of DNA-based structures [125] and the recognition of such structures in solution. Thin polymer layer able to recognize double-stranded DNA (dsDNA) was developed by using 2-vinyl-4,6-diamino-1,3,5-triazine (VDAT) as a functional monomer for the formation of DNA-imprinted polymer [195]. A molecularly imprinted electrochemoluminescence sensor for ultrasensitive HIV-1 gene detection using EuS nanocrystals as luminophore was developed [190]. Methylene blue imprinted polyvinyl pyridine polymer modified carbon paste electrodes were applied for the electrochemical monitoring of DNA [196].

During the development of protein sensors molecularly imprinted polypyrrole based sensor for direct detection of bovine leukemia virus glycoproteins was designed in our group [186]. Human serum albumin (HSA) imprinted o-phenylenediamine and hydroquinone (HQ) based copolymer, which was electrochemically deposited on a gold electrode orderly pre-modified by gold nanoparticles and polythionine-methylene blue was applied for the determination of HSA in urine [197]. Surface molecularly imprinted polydopamine films were applied for the determination of immunoglobulin *G* [198]. Electrosynthesized surface-imprinted PEDOT/PSS-based microrods were applied for selective protein recognition [187]. Synthetic receptors, which were comprising of highly selective aptamer-lined pockets formed within electropolymered dopamine, deposited on metal–oxide–semiconductor field-effect transistor (MOSFET) was dedicated for the detection of prostate specific antigen in human blood plasma were developed [199]. Electrochemically formed molecularly imprinted polypyrrole/(carbon nanotube) composite was applied in electrochemical sensor for the determination of S-ovoalbumin in egg white [200]. Electrochemically deposited molecularly imprinted poly–*o*–phenylenediamine was applied for direct electrochemical determination of myoglobin [201]. Electrosynthesized molecularly imprinted polyscopoletin nanofilms were used for the detection of human serum albumin [202]. Sensor based on poly-scopoletin imprinted by cytochrome *C*, deposited on 11-Mercaptoundecanoic acid (MUA) based self-assembled monolayer was applied for direct detection of cytochrome *C* [203]. Lysozyme-imprinted hydroxyethyl acrylate and ethylene glycol dimethacrylate based copolymer microspheres were applied for the determination of lysozyme [204]. *N*-Acetylneuraminic acid molecularly imprinted poly(2-hydroxyethyl methacrylate-*N*-methacryloyl-(L)-histidin-Cu(II)) has been synthesized by radical polymerization and applied for site recognition of highly biologically active protein–ceruloplasmin [205]. It should be noted that very recently portable molecularly imprinted poly-m-phenylenediamine based electrochemical sensor for the detection of SARSCoV-2 antigen was developed [107], which is much cheaper in comparison to recently developed ellipsometric COVID-19 diagnosis techniques based on SARSCoV-2 nucleocapsid protein and specific antibody complex formation [206].

Alpha-fetoprotein-imprinted ortho-polydopamine (o-polydopamine) electro-deposited on AuNPs-based film, in which alpha-fetoprotein was temporarily covalently immobilized before electrochemical polymerization of dopamine, was developed [207]. Differential pulse voltammetry was applied for the determination of analytical signal, where the peak current has decreased with the concentration of α-fetoprotein increasing. This MIP sensor was characterised by long linear range within 0.001 ng/mL and 800 ng/mL and α-fetoprotein detection limit of 0.8 pg/mL. Another affinity sensor was based on a acrylamide/*N*,*N*_0_-methylenebisacrylamide copolymers, which were imprinted by different analytes: (i) prostate-specific antigen and (ii) myoglobin [208]. Before electrochemical polymerization of dopamine both target proteins were covalently immobilized on the surface of 3,30-dithiodipropionic acid di(*N*-hydroxysuccinimide ester), which was pre-deposited on substrate before the immobilization. Then acrylamide/*N*,*N*_0_-methylenebisacrylamide copolymer based MIPs were formed on the surfaces of electrodes, which were applied for the determination of prostate-specific antigen and myoglobin in human urine and blood serum. Hemoglobin imprinted polyacrylamide membrane was reported [209]. O-phenylenediammine was applied for the determination of protein troponin *T* [210], which is a specific biomarker for myocardial tissue that is used as cardiac biomarker for early cardiac disease diagnosis.

It is interesting to note that some researchers are reporting that polypyrrole was successfully imprinted by spores of *Bacillus cereus* [211], and even by bacteria such as *Escherichia coli* [212]. Hence, these and some other MIP-based investigations illustrate that MIPs are suitable for the design of sensors dedicated for the determination of infectious diseases [213], viruses [214], spores [211], and bacteria [212,215,216] imprinted MIPs based sensors.

Numerous abovementioned researches were dedicated for the application of polypyrrole, overoxidized polypyrrole [1,80,98] and/or phenylenediamine-derivatives [106] in MIP-based sensors, but another for different technological purposes very frequently used conducting polymer–polyaniline (PANI) is still rarely applied for the development of molecular imprinting-based sensors. In one report molecularly imprinted PANI was applied for the determination of antibiotic azithromycin at low concentrations, which were below 0.1 nM [217].

Selection of the most suitable polymeric matrix for the formation of MIP-based layer is very important task during the fabrication of MIP-technology based affinity sensors [218], the newly designed MIP should have capability to interact with target electrostatically, via van der Waals forces, formation of hydrogen bonds, π-π interactions and/or establishing of some ‘hydrophobic interactions’ [219]. Here addressed interactions enable reversible formation/dissociation of complex between analyte and imprints formed in the MIP-based layer [174,220,221]. During the realization of some strategies applied in the design of MIP-based sensors some additional functional groups, which are able to form complexes with target, can be incorporated into the polymeric structure [175]. It is important to note that formed complex between an imprinted template and MIP-based matrix should be not very strong, because then it will be hard to remove template from the matrix, because imprinted template molecules usually are simply “washed out” by usual solvents such as water [173]. One of the main advantages of MIP-based sensors is the fact that in comparison with antibodies or receptors based sensors they are more stable at a broad temperature interval. Dependently on applied monomers MIPs can be divided into several types; the simplest MIPs are based on single monomer, which is applied in the formation of polymeric matrix [173] such sensors can be based on the conducting polymers, which if necessary, can be overoxidized to create functional groups that are able more efficiently interact with the target analyte [80,85], e.g., Ozkan’s research group has applied overoxidized Ppy (overoxidized-Ppy), which was involved into carboxylic acid functionalized multi-walled carbon nanotubes and overoxidized polypyrrole modified (overoxidized-Ppy/MWCNTs-COOH/GCE) glassy carbon electrodes by cyclic and adsorptive stripping differential pulse voltammetric techniques for selective determination of Pemetrexed [222] and Ppy/GCE electrode for the determination of Adefovir [223].

## 7. Physicochemical Methods Used for the Determination of Analytical Signal by MIPs Based Sensors

A vast number of research illustrates the applicability of electrochemical methods in MIP-based sensors. The determination of target-binding generated signal can be performed directly, if the target is electrochemically active, and/or the determination can be performed indirectly by the modulation of diffusional permeability of a redox probe through MIP-based layer. However, in an indirect way registered overall signal includes effects from all nonspecific interactions. Therefore, MIP-based methods for the determination of redox-active low-molecular-weight analytes and the application of some redox-active metalloproteins enable a more accurate direct determination of target interaction with MIPs using determination of electrochemical activity [224] or enzymatic (e.g., acetylcholinesterase [225], tyrosinase [226], glucose oxidase [227], creatine kinase [228], hexameric heme protein [229], cytochrome P450 [230], laccase [231], horseradish peroxidase [232,233,234], microperoxidase [232], lactoperoxidase [232], and hemoglobin [232] catalytic activity of target molecules. Pt/Cu bimetallic nanoparticles modified by MIP-based on poly (styrene sulfonate) functionalized by graphene, which showed peroxidase-like activity to catalyze the oxidation of 3,3′,5,5′-tetramethylbenzidine (TMB) in the presence of H_2_O_2_, was applied for the determination of heterocyclic isoflavone–puerarin [235]. Trypsin imprinted molecularly imprinted water-soluble methacryloylaminobenzamidine micro-gels were applied as inhibitors of this enzyme, because molecularly imprinted sites were selectively bounded in close proximity of the substrate recognition site of trypsin. [236].

Several very different mechanisms of the “gate effect” can be successfully applied for analyte quantification by MIPs-based electrochemical affinity sensors: (i) The simplest mechanism involves structural rearrangement of MIP-based layer by shrinking or swelling caused by binding of analyte within imprinted structure, such changes of MIP-structure influence the diffusion rate of ions or/and the redox probe through the film and, therefore, they can be easily detected by many different electrochemical methods [2,185,237]; (ii) The next mechanism is based on the charged state of the MIP-forming polymer, e.g., during the interaction with target the accumulation positively/negatively charged ions is restricting the diffusion of a oppositely charged ions and/or redox probes [2,185,238]. In addition, the electronic structure of the MIP-forming polymer can vary due to removing/entrapping of analyte from/into the MIP structure, thus changing the conductivity of MIP-based layer [2,185,237], acting MIP-based modified gate electrode was incorporated within organic electrochemical transistor and applied in the design of sensor suitable for the determination of ascorbic acid [239].

Quartz crystal microbalance (QCM) is a method, which can be easily coupled with electrochemistry when it is applied in electrochemical QCM (EQCM) setup [23,93]. QCM-based methods were applied for the determination: (i) of low molecular weight analytes [240,241], including histamine [242], naproxen [243], ibuprofen [244], *S*-propranolol [245]; (ii) proteins [246,247,248,249], ribonuclease A [250], oxidized-low-density lipoprotein in blood serum [251] and trypsin [252]; (iii) DNA and [249,253]. More advanced QCM-based techniques such as electrochemical QCM (EQCM) [23,93,254] and QCM with dissipation (QCM-D) [255] were also applied for the determination of analytical signals generated by molecularly imprinted and not imprinted CPs-based sensors.

As it was presented above various electrochemical detection methods used for the determination of analytical signal [23,93,256] other analytical signal determination methods such as photoluminescence [126,140,257] and QCM [23,93] are used for MIP-based molecular recognition. In addition to above mentioned methods, it was demonstrated that surface plasmon resonance can be applied for the evaluation of analytical signal by MIP-based sensors, e.g., theophylline-imprinted poly-methacrylic acid based SPR sensor has been reported [258]. Very attractive area in the development of molecularly imprinted materials and new methods for the determination of analytical signals is based on the application of combination of photonic crystals with molecularly imprinted polymers [259]. It was demonstrated that some liquid crystals with a different optical birefringence can be applied in MIP-based sensor design, which are suitable for the determination of proteins [260].

## 8. Towards Implantable Affinity Sensors Based on Biocompatible Polymers

Biocompatibility of sensor structures is an extremely important issue during the design of implantable affinity-based sensors and biosensors. However, in the most scientific papers compatibility of polymers and especially of conducting polymers is investigated just with simple biological structures such as proteins, e.g., the ability of entrapped enzymes to retain their catalytic activity [10,33,41,46,47,48]. However, such investigations are not presenting the real biocompatibility of these polymers, because the evaluation of cellular response to any implantable/attachable material is essential for all biomedical applications [261]. For this reason, experiments on laboratory animals or on actual cell lines are required to evaluate the biocompatibility of the most conducting polymers. Hence, in some research we have demonstrated that some CPs including Ppy show sufficient compatibility towards immobilized proteins [10,12,33,41,46,47,48]. Our and some other researches have illustrated that Ppy-based polymeric structures are biocompatible to steam cells [49,50] and neuronal cells [262] and CPs are just not significantly influencing the immune system of mammalians [51]. It was determined that Ppy-based particles do not affect hematological parameters of immune system [51]. However, some toxicity of Ppy-based nanoparticles on stem cells derived from bone marrow were observed [49]. We also investigated the toxicity of Ppy-based nanoparticles towards mouse hepatoma cell line (MH-22A), human T lymphocyte Jurkat cells and primary mouse embryonic fibroblasts (MEF), which was very low [40]. Conducting polymer-based hydrogels have good biocompatibility, which is determined by the presence of significant amount of water in their structure [263] and, if it is necessary, it can be improved by the addition of biocompatible polymers like chitosan [264] or other biocompatible materials [265,266,267]. It was demonstrated that chitosan-based structures, which can be modified in many different ways, are able to advance selectivity and some other analytical properties of molecularly imprinted polymers [268]. Therefore, some CP-based hydrogels were used for the immobilization of living cells during the formation of scaffolds [269,270] that were applied for transplantation [271] and for many other biomedical purposes [272,273,274,275]. Very good biocompatibility of conducting polymer polypyrrole [49,50,51] and some other polymers enables to apply them in the future for the design of implantable/attachable [276], wearable [277], and other [278,279] sensors.

## 9. Conclusions

Conducting and many other polymers are offering many analytical and/or technological advantages and, therefore, they are finding applications in various analytical and bioanalytical systems. The ability to design molecularly imprinted polymers enables to create such artificial structures that can replace some natural biological structures such as DNA-aptamers or biological recognition exhibiting proteins (including antibodies and receptors). Various polymerization methods can be applied for the formation of molecularly imprinted polymers, but electrochemical deposition of conducting polymer some other polymers that can be electrochemically deposited on electrodes are the most promising, because electrochemical methods enable to adjust the most suitable electropolymerization conditions. Therefore, a variety of electrochemically deposited conducting polymers with very different properties can be designed and among many other polymers these CPs are offering probably the most interesting technological possibilities for the design of MIPs based sensors. Especially efficiently overoxidation of some conducting polymers (e.g., polypyrrole) can be exploited for the removal of template, regeneration of molecularly imprinted polymer-based layer and what is the most important—for the establishment of oxidized groups that are providing selectivity towards molecularly imprinted analytes. It should be noted that some MIP-based sensors are robust and are operating at room temperature, are providing high selectivity and sensitivity. Electrochromic properties of some conducting polymers are already used in some affinity sensors for the determination of metal ions, therefore, electrochromism eventually can be also exploited in the design of signal transduction systems of some MIP-based sensors. Many polymerization methods can be applied for the formation of conducting polymer-based layers, probably the most unique and well controllable polymerization methods are based on electrochemical techniques, because these techniques are providing abilities for the most efficient control and modification of formed sensing layers. Hence, the properties of CPs can be tuned in many different ways. Polypyrrole among many other conducting polymers is the most frequently used in the design of MIP-based sensors and due to easy synthesis from water-based solutions and easily achievable overoxidation, therefore, Ppy has a great potential for the development or cheap, sensitive and robust sensors based on artificial receptor-like structures. Electrochemical deposition of conducting polymer-based layers enables us to design sensing coatings that are having very different physical properties. Therefore, arrays of such electrochemical sensors can be developed, in these arrays individual sensors will differently respond towards similar mixtures of analytes, and registered response patterns can be analyzed using multivariate analysis of variance (MANOVA) algorithms. Some CPs are showing good biocompatibility, therefore, they have great potential to be applied in the design of implantable sensors and other biomedical devices.

## Data Availability

Not applicable.
